# *Candida albicans*-stimulated hematopoietic stem and progenitor cells generate trained neutrophils with enhanced mitochondrial ROS production that defend against infection

**DOI:** 10.1371/journal.ppat.1013170

**Published:** 2025-05-13

**Authors:** María Sobén, Paula Guerrero, Andrea Guiu, Alberto Yáñez, María Luisa Gil

**Affiliations:** Instituto de Biotecnología y Biomedicina (BIOTECMED) and Departamento de Microbiología y Ecología, Facultad de Ciencias Biológicas, Universitat de València, Burjassot, Spain; Rutgers New Jersey Medical School, UNITED STATES OF AMERICA

## Abstract

Central trained immunity, induced via reprogramming of hematopoietic stem and progenitor cells (HSPCs), mediates sustained heightened responsiveness of mature myeloid cells to secondary challenges. We have previously demonstrated that HSPCs use TLR2 and Dectin-1 to sense *Candida albicans* to induce the production of trained monocytes/macrophages to fight against secondary infection. Neutrophils play an important role in innate immunity and are critical for clearance of *C. albicans.* In this work, we used an *in vitro* model of mouse HSPC differentiation to investigate the functional phenotype of neutrophils derived from HSPCs exposed to various PAMPs and *C. albicans* cells. We found that neutrophils derived from HSPCs stimulated by a TLR2 agonist exhibit reduced inflammatory cytokine production (tolerized neutrophils) whereas neutrophils generated from a Dectin-1 agonist or *C. albicans* stimulated HSPCs produce higher amounts of cytokines (trained neutrophils). We further demonstrated that a transient exposure of HSPCs to live *C. albicans* cells is sufficient to induce a trained phenotype of the neutrophils they produce in a Dectin-1- and TLR2-dependent manner. These trained neutrophils exhibited higher phagocytosis and microbicidal capacity than control neutrophils. Additionally, their adoptive transfer was sufficient to reduce fungal burden during invasive candidiasis. Mechanistically, we demonstrated that trained neutrophils use mitochondrial ROS (mtROS) to enhance their ability to kill *C. albicans* cells, as they produce higher amounts of mtROS and scavenging mtROS with MitoTEMPO attenuated their yeast-killing ability to match that of control neutrophils. Altogether, these data suggest that infection-experienced HSPCs contribute to trained immunity by providing a source of trained neutrophils with enhanced antimicrobial activity which may confer prolonged protection from infection. The tailored manipulation of this mechanism might offer new therapeutic strategies for controlling fungal infections by harnessing neutrophils.

## Author summary

Invasive candidiasis is an increasingly frequent cause of serious and often fatal infection. Understanding host defense is essential to design novel therapeutic strategies to boost immune protection against *Candida albicans*. Neutrophils are innate immune cells that are crucial for controlling *C. albicans* infection. In this work, we delve into new concepts that have arisen over the last years: (i) trained innate immunity extends to hematopoietic stem and progenitor cells (HSPCs), a process called central trained immunity and (ii) neutrophils may contribute to the protective effects of trained immunity, as already described for monocytes and macrophages. We demonstrate that *C. albicans*-stimulated HSPCs generate trained neutrophils with an increased cytokine production, as well as with an improved ability to internalize and kill yeast cells. Furthermore, the adoptive transfer of trained neutrophils defends mice against infection. Mechanistically, we demonstrate that trained neutrophils use mitochondrial ROS (mtROS) to enhance their ability to kill *C. albicans* cells. Taken together, these data indicate that HSPCs can sense pathogens during infection and contribute to protect the host by generating trained neutrophils. Targeting this mechanism may provide a therapeutic strategy to boost neutrophil function and treat or prevent infectious diseases.

## Introduction

Invasive fungal infections by *Candida albicans* are the most prevalent of the opportunistic mycoses. Systemic candidiasis is an increasingly frequent cause of life-threatening infections in hospitalized and immunosuppressed patients. Phagocytes, such as neutrophils, monocytes, macrophages, and dendritic cells are crucial for host defence against invasive candidiasis. During infection, these myeloid cells can detect the microorganism by using several pattern recognition receptors (PRRs), including different members of the Toll-like receptor (TLR) and C-type lectin receptor (CLR) families. They are responsible for releasing proinflammatory cytokines to recruit and activate other leukocytes, as well as for microbial killing and antigen processing and presentation to initiate the adaptive immune response [1].

Immune memory is not an exclusive feature of adaptive immunity; innate immune cells can also display some memory characteristics and respond in a different way upon re-exposure to the same or heterologous stimuli. For instance, exposure of monocytes and macrophages to *C. albicans* enhanced their subsequent inflammatory response to stimulation (trained immunity), while TLR2 and TLR4 ligands confer a reduced response to macrophages (tolerance). The mechanisms leading to this functional reprogramming of cells include metabolic changes and epigenetic remodelling [2].

This concept of innate immune memory extends beyond short-lived mature myeloid cells to hematopoietic stem and progenitor cells (HSPCs), explaining the described long-term memory [3,4]. In this context, we have demonstrated that HSPCs exposed to a TLR2 ligand or Dectin-1 agonists produce soluble factors that act in a paracrine manner on unexposed HSPCs, leading to the generation of tolerized or trained macrophages respectively [5–8].

Trained immunity may be beneficial for the host as it confers protection to secondary infection. It has been described in several models that trained monocytes/macrophages protect against heterologous infections, including candidiasis [9]. However, the potential role of trained neutrophils in protection during candidiasis remains unexplored, despite the essential role of this cell type in protection against *C. albicans* infection. Neutrophils are the first cells to be recruited to the site of infection and are the most effective killers of *C. albicans*. In fact, neutropenia is a major risk factor for invasive candidiasis [10,11].

Although neutrophils were considered to be a homogeneous population unable to adapt to changing environments, recent evidence indicates that neutrophils are heterogeneous and can respond to environmental changes with significantly altered gene expression. Trained neutrophils have been previously described in some models [12,13]. In fact, pre-treatment of mice with β-glucan resulted in a tumour growth reduction, which was associated with transcriptomic and epigenetic rewiring of granulocyte progenitors and the generation of trained neutrophils [14]. Furthermore, BCG vaccination in humans induced trained immunity in neutrophils, characterized by increased expression of activation markers, and greater responsiveness to heterologous stimulation [15]. Lastly, neutrophils and monocytes had enhanced anti-microbial activity, and mice were protected from listeriosis nine weeks after induction of training with β-glucan [16].

In this study, we demonstrate that PRR signalling in HSPCs determines the antifungal phenotype of the neutrophils they produce. A transient exposure of HSPCs to live *C. albicans* cells, prior to differentiation, is sufficient to induce a trained phenotype to the differentiated neutrophils in a Dectin-1 and TLR2-dependent manner. Our work shows that trained neutrophils exhibit an increased cytokine production, as well as an improved ability to kill microorganisms. Moreover, the adoptive transfer of trained neutrophils is sufficient to reduce fungal burden during invasive candidiasis. Mechanistically, we demonstrate that trained neutrophils use mitochondrial ROS (mtROS) to enhance their ability to kill *C. albicans* cells. Taken together, these data indicate that HSPCs can sense pathogens during infection and contribute to protect the host by generating trained neutrophils that produce higher levels of cytokines and use mtROS for their enhanced fungicidal activity.

## Results

### Exposure of HSPCs to TLR2 and Dectin-1 agonists modulates cytokine production by the neutrophils they produce

For *in vitro* HSPC differentiation to neutrophils, lineage negative (Lin^–^) cells were cultured with granulocyte-colony stimulating factor (G-CSF) and IL-3. After 8 days of culture, non-adherent cells were harvested and analyzed by flow cytometry (S1A Fig). In these conditions, the non-differentiated cells (c-Kit^+^) represented roughly 1%, whereas most of cells differentiated into myeloid CD11b^+^ cells (88%). The percentage of neutrophils (CD11b^+^ Ly6G^+^) represented approximately 64% of the myeloid CD11b^+^ population.

**Fig 1:**
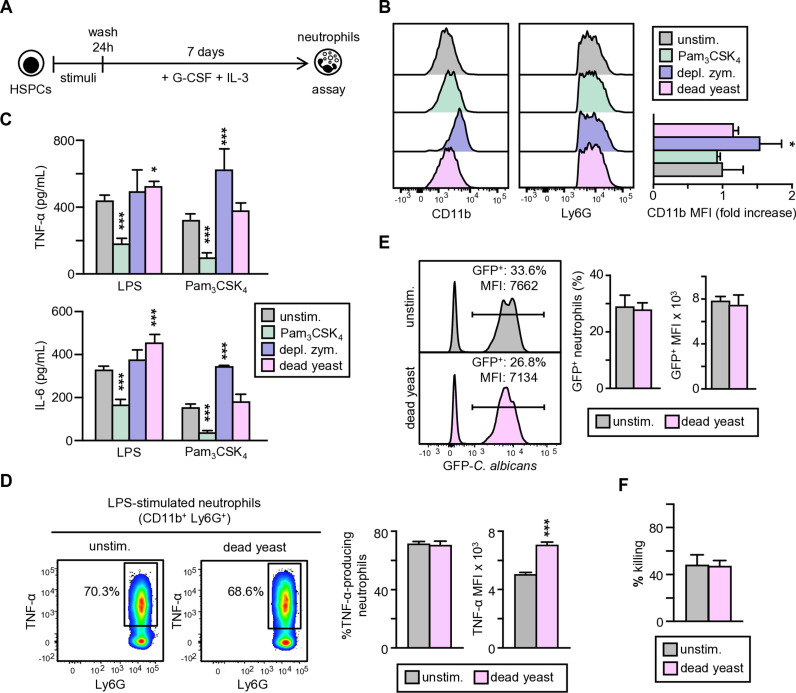
Exposure of HSPCs to TLR2 and Dectin-1 agonists modulates the phenotype of the neutrophils they produce. (A) Schematic protocol of Lin^–^ cell stimulation and differentiation (created using Power Point as a tool). Lin^–^ cells were cultured in medium containing SCF, G-CSF and IL-3 to induce neutrophil differentiation, in the presence or absence (unstimulated) of different stimuli during the first 24 h: Pam_3_CSK_4_ (1 μg/ml), depleted zymosan (10 μg/ml), and dead yeasts at a 1:5 ratio (murine cell:yeast). (B) After 8 days of culture non-adherent cells were antibody labelled and neutrophils were gated as c-Kit^–^ CD11b^+^ Ly6G^+^ cells. The expression of CD11b and Ly6G was measured (histograms are from 1 representative experiment), and fold increase of CD11b MFI measurements are means ± SD of pooled data from 3 independent experiments. (C) Purified neutrophils were stimulated with LPS or Pam_3_CSK_4_ and TNF-α and IL-6 production was measured by ELISA in supernatants. (D) TNF-α production was measured by intracellular flow cytometry upon LPS stimulation. Dot-plots indicating cytokine-producing neutrophils are shown (1 representative experiment), as well as the % and MFI of TNF-α-producing neutrophils. For the ELISA measurements at least triplicate samples were analyzed and expressed as means ± SD. Data from one representative experiment of two is shown. For intracellular cytokine measurement pooled data from three experiments are shown. (E) Phagocytosis assay. Neutrophils were challenged with GFP-*C. albicans* yeasts at a 1:7.5 ratio (murine cell:yeast) for 30 min. Afterward, neutrophils were gated as Ly6G^+^ cells and the extent of phagocytosis was assessed as the percentage of GFP^+^ cells (% of phagocytosis) as well as the MFI of green fluorescence. Data are expressed as means ± SD from 3 independent experiments. (F) Fungicidal activity of neutrophils. Neutrophils were challenged with viable PCA2 yeasts at a 1:3 ratio (murine cell:yeast) for 1.5 h. After incubation, samples were diluted, plated on Sabouraud dextrose agar and incubated overnight at 37 °C; CFUs were counted and killing percentages were determined as indicated in materials and methods. Triplicate samples were analyzed in each assay. Data are presented as means ± SD of pooled data from 3 independent experiments. Statistical significance was assessed by the Student *t* test and the 1-way analysis of variance (ANOVA) followed by Dunnett test for multiple comparisons (*P < 0.05, and ***P < 0.001).

Next, we investigated the consequences of *in vitro* exposure of HSPCs to pathogen associated molecular patterns (PAMPs) by comparing the phenotype of the neutrophils they produce. For that, Lin^–^ cells were cultured to induce neutrophil differentiation in the presence or absence of different PRR agonists during the first 24 h: Pam_3_CSK_4_ (which only activates TLR2), depleted zymosan (a pure Dectin-1-activating *Saccharomyces cerevisiae* cell wall preparation), and inactivated *C. albicans* yeasts (which activates several PRRs, but principally TLR2 and Dectin-1) (Fig 1A). In all cases, the generated neutrophils (CD11b^+^ Ly6G^+^) expressed similar levels of Ly6G, whereas the neutrophils generated by depleted zymosan-exposed HSPCs exhibited increased expression of CD11b, suggesting enhanced activation and antimicrobial activity (Fig 1B).

Neutrophils were purified, counted and equal numbers of cells were plated and stimulated with TLR agonists to assess their ability to produce proinflammatory cytokines (Fig 1C). The production of TNF-α and IL-6 in response to LPS or Pam_3_CSK_4_ was significantly diminished in neutrophils generated from HSPCs exposed to Pam_3_CSK_4_, compared to neutrophils generated from unstimulated HSPCs (control neutrophils). Interestingly, the secretion of TNF-α and IL-6 in response to Pam_3_CSK_4_ was increased in neutrophils generated from HSPCs exposed to depleted zymosan, and the production of cytokines in response to LPS was increased in neutrophils generated from *C. albicans*-activated HSPCs. The higher production of cytokines measured by ELISA might be explained by higher numbers of cytokine-producing cells or by a reprogrammed neutrophil function for a trained response. To assess this, we measured intracellular TNF-α production at the single-cell level by flow cytometry (Fig 1D). The percentage of TNF-α-producing neutrophils were equal in response to LPS, whereas the mean fluorescence intensity (MFI) of the cytokine-producing neutrophils showed an enhanced production of TNF-α by each individual cell in neutrophils derived from *C. albicans*-exposed HSPCs compared to control neutrophils.

To further characterize the antifungal function of neutrophils derived from *C. albicans*-exposed Lin^–^ cells, we measured their ability to internalize and kill yeast cells. First, neutrophils were challenged with GFP-*C. albicans* yeasts, and phagocytosis of the yeast cells by neutrophils (gated as Ly6G^+^ cells) was analyzed by flow cytometry and expressed as the percentage of cells that contain internalized yeasts, as well as the MFI, which indicates the extent of phagocytosis per cell (Fig 1E). The *C. albicans* activation of HSPCs changed neither the percentage nor the extent of phagocytosis of derived neutrophils. Finally, neutrophils were challenged with viable yeasts in order to determine their fungicidal activity (Fig 1F). *In vitro* differentiated neutrophils were able to kill a significant percentage of yeasts: CFUs after the coculture were reduced a 40%. Exposure of the progenitors to inactivated *C. albicans* cells did not changed the fungicidal activity of the neutrophils derived from them.

These results indicate that exposure of HSPCs to PRR agonists may alter the ability of the derived neutrophils to produce proinflammatory cytokines. TLR2 ligands cause a reduction in cytokine production while particulate Dectin-1 agonist and inactivated yeasts provoke an increased response. However, these trained neutrophils do not alter their ability to internalize and kill yeast cells.

### Exposure of HSPCs to live cells of *C. albicans* generates trained neutrophils in a Dectin-1- and TLR2-dependent manner

**Fig 2:**
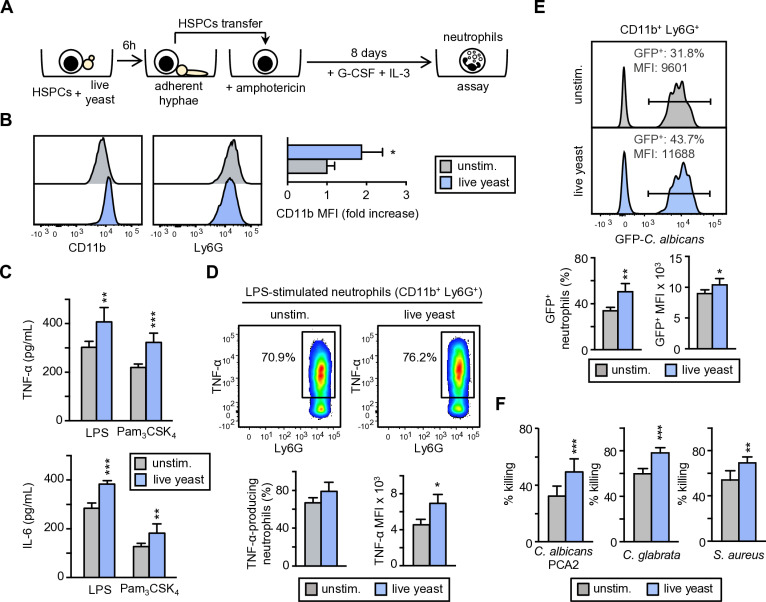
Exposure of HSPCs to live cells of *C. albicans* confers a trained phenotype to the neutrophils derived from them. (A) Schematic protocol of Lin^–^ cell stimulation and differentiation (created using Power Point as a tool). Lin^–^ cells were cultured in the presence or absence (unstimulated) of live *C. albicans* cells at 1:0.5 ratio (progenitor:yeast) for 6 hours, and then amphotericin B (0.5 μg/ml) was added. Lin^–^ cells were transferred to a new plate and cultured with SCF, G-CSF and IL-3 for 8 days to obtain neutrophils. (B) After 8 days of culture non- adherent cells were antibody labelled and neutrophils were gated as c-Kit^–^ CD11b^+^ Ly6G^+^ cells. The expression of CD11b and Ly6G was measured (histograms are from 1 representative experiment), and fold increase of CD11b MFI measurements are means ± SD of pooled data from 3 independent experiments. (C) Purified neutrophils were stimulated with LPS or Pam_3_CSK_4_ and TNF-α and IL-6 production was measured by ELISA in supernatants. (D) TNF-α production was measured by intracellular flow cytometry upon LPS stimulation. Dot-plots (1 representative experiment), indicating cytokine-producing neutrophils are shown, as well as the % and MFI of TNF-α-producing neutrophils. For the ELISA measurements at least triplicate samples were analyzed and expressed as means ± SD. Data from one representative experiment of two is shown. For intracellular cytokine measurement pooled data from three experiments are shown. (E) Phagocytosis assay. Neutrophils were challenged with GFP-*C. albicans* yeasts at a 1:7.5 ratio (murine cell:yeast) for 30 min. Afterward, neutrophils were gated as Ly6G^+^ cells and the extent of phagocytosis was assessed as the percentage of GFP^+^ cells (% of phagocytosis) as well as the MFI of green fluorescence. Data are expressed as means ± SD from 4 independent experiments. (F) Microbicidal activity of neutrophils. Neutrophils were challenged with viable PCA2 or *C. glabrata* yeasts at a 1:3 ratio (murine cell:yeast) for 1.5 h, or alternatively with viable *S. aureus* bacteria at a 1:20 ratio (murine cell:bacteria) for 50 min. After incubation, samples were diluted, plated on Sabouraud dextrose agar for fungal strains or on Mueller-Hinton agar for bacteria and incubated overnight at 37 °C; CFUs were counted and killing percentages were determined as indicated in materials and methods. Triplicate samples were analyzed in each assay. Data are presented as means ± SD of pooled data from 2 or 3 independent experiments. Statistical significance was assessed by the Student *t* test and the 1-way analysis of variance (ANOVA) followed by Dunnett test for multiple comparisons (*P < 0.05, **P < 0.01, and ***P < 0.001).

The consequences of exposing HSPCs to inactivated yeasts may be different from those of exposing them to live yeasts, which express several virulence factors, including the yeast-to-hypha transition, candidalysin and metabolite secretion among others. Therefore, we next investigated the functional consequences of activation by live *C. albicans* cells, at the HSPC stage, prior to neutrophil differentiation (Fig 2). For these experiments, Lin^–^ cells were cultured in the presence or absence of live *C. albicans* cells at a 1:0.5 ratio (progenitor/yeast) for 6 h (Fig 2A). Under these experimental conditions, i.e., a low number of fungal cells, presence of serum, and at 37 °C, most of the yeasts performed the conversion from budding yeast to filamentous growth form. After 6 h of coculture, amphotericin B was added to stop fungal growth. Taking advantage of the high adhesion of hyphae to plastic, Lin^–^ cells were transferred to a new plate leaving the hyphae adhered to the original plate and were cultured with IL-3 and G-CSF for 8 days to obtain neutrophils. All generated neutrophils expressed similar levels of Ly6G, whereas the neutrophils generated by live *C. albicans*-exposed HSPCs exhibited higher expression of CD11b than control neutrophils, indicating improved antimicrobial activity (Fig 2B). Equal numbers of purified neutrophils, derived from unexposed or live yeast-exposed HSPCs, were plated to examine their functional phenotype. Results showed that the production of TNF-α and IL-6 in response to LPS or Pam_3_CSK_4_ was significantly increased in neutrophils derived from HSPCs exposed to live *C. albicans* cells (Fig 2C), and this was due to an enhanced production of TNF-α by each individual cell; that is, these cells exhibited a trained phenotype (Fig 2D).

Next, we measured the ability of these trained neutrophils to internalize and kill yeast cells. The activation of HSPCs by *C. albicans* live cells increased both, the percentage and the extent of phagocytosis of derived neutrophils (Fig 2E). Moreover, exposure of the progenitors to live *C. albicans* cells enhanced the ability to kill PCA2-*C. albicans* yeasts of the neutrophils derived from them (Fig 2F). To further confirm the increased fungicidal activity of trained neutrophils, we also tested their ability to kill yeasts of *Candida glabrata*. While *C. albicans* is the most frequently detected species in fungal invasive infection, non-albicans *Candida* species are increasingly relevant, particularly in high-risk populations, with *C. glabrata* being the most prominent. Moreover, *C. glabrata* is unable to form pseudohyphae; it exists as blastoconidia independently of environmental conditions [17] making this specie especially suitable and appropriate for killing assays. The fungicidal activity against *C. glabrata* was similar to that against *C. albicans*, confirming the higher fungicidal activity of neutrophils generated from live yeast-exposed HSPCs. Finally, to evaluate whether this increased killing ability extends beyond fungal pathogens, we tested the capacity to kill the bacterial pathogen *Staphylococcus aureus*. We found that neutrophils derived from HSPCs stimulated with live *C. albicans* cells had significantly higher bacterial killing rates when compared to neutrophils derived from control HSPCs (Fig 2F).

**Fig 3:**
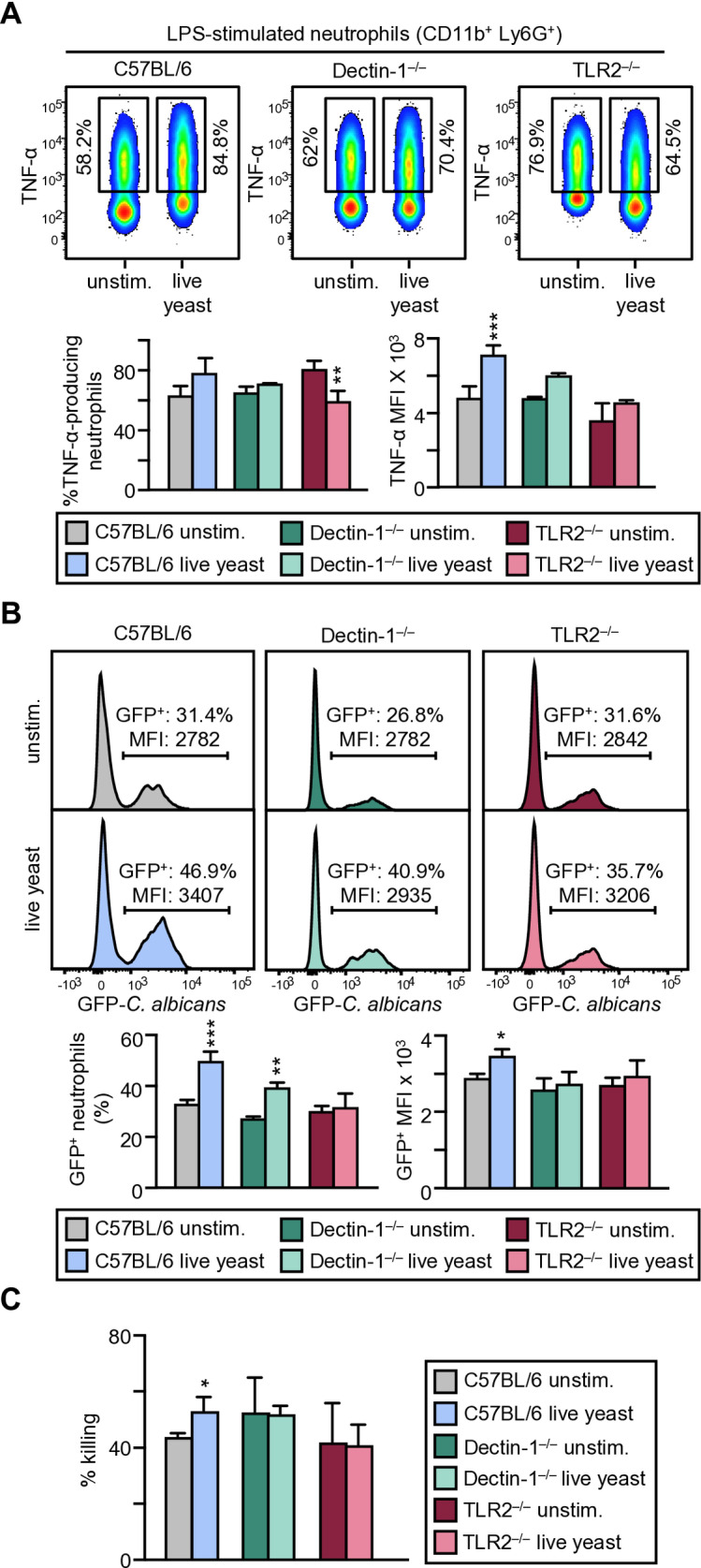
The generation of trained neutrophils in response to live cells of *C. albicans* is dependent on both TLR2 and Dectin-1 signalling in HSPCs. Lin^–^ cells from wild-type, Dectin 1^–/–^ or TLR2^–/–^ mice were cultured in the presence or absence of live *C. albicans* cells and then differentiated to neutrophils as indicated in the schematic protocol in Fig 2A. (A) TNF-α production was measured by intracellular flow cytometry upon LPS stimulation. Dot-plots (1 representative experiment), indicating cytokine-producing neutrophils are shown, as well as the % and MFI of TNF-α-producing neutrophils. Data are expressed as means ± SD of pooled data from 3 independent experiments. (B) Phagocytosis assay. Neutrophils were challenged with GFP-*C. albicans* yeasts at a 1:7.5 ratio (murine cell:yeast) for 30 min. Afterward, neutrophils were gated as Ly6G^+^ cells and the extent of phagocytosis was assessed as the percentage of GFP^+^ cells (% of phagocytosis) as well as the MFI of green fluorescence. Data are expressed as means ± SD of pooled data from 3 independent experiments. (C) Fungicidal activity of neutrophils. Neutrophils were challenged with viable PCA2 yeasts at a 1:3 ratio (murine cell:yeast) for 1.5 h. After incubation, samples were diluted, plated on Sabouraud dextrose agar and incubated overnight at 37 °C; CFUs were counted and killing percentages were determined as indicated in materials and methods. Triplicate samples were analyzed in each assay. Data are presented as means ± SD of pooled data from 3 independent experiments. Statistical significance was assessed by the 1-way analysis of variance (ANOVA) followed by Dunnett test for multiple comparisons (*P < 0.05, **P < 0.01, and ***P < 0.001).

These results prompted us to investigate whether TLR2 and/or Dectin-1 signalling in HSPCs is playing a role in our *in vitro* model using live yeasts as stimuli (Fig 3). Lin^–^ cells from TLR2^–/–^ or Dectin-1^–/–^ mice exposed to live *C. albicans* cells gave rise to neutrophils that produced similar levels of TNF-α as control neutrophils (Fig 3A). In the absence of TLR2 or Dectin-1 signalling in the progenitors, the challenge with live yeasts did not induce an increase in the ability of neutrophils to internalize and kill *C. albicans* cells (Fig 3B and 3C). Phagocytosis of yeasts by Dectin-1^–/–^ neutrophils was slightly reduced in comparison with phagocytosis by WT neutrophils [26.8% versus 31.4% (Fig 3B)], but the difference was not statistically significant. Probably, other PRRs are playing a role in the internalization of yeasts upon recognition of other PAMPs in our experimental conditions. The challenge with live yeasts induced a slight increase in the ability of Dectin-1^–/–^ neutrophils to internalize yeasts (in the % not in the MFI) although this increase was lower than the increase in trained wild-type neutrophils. In the absence of TLR2 signalling in the progenitors, the challenge with live yeasts did not induce an increase in the ability of neutrophils to internalize *C. albicans* cells (Fig 3B). Besides, in the absence of TLR2 or Dectin-1, the challenge with live yeasts did not induce an increase in the ability of neutrophils to kill *C. albicans* cells (Fig 3C). Overall, these results indicated that in this model of exposure to live *C. albicans* cells, the generation of trained neutrophils is dependent on both TLR2 and Dectin-1.

These results suggest that HSPCs can sense live *C. albicans* cells during infection directly in a TLR2 and Dectin-1 dependent manner to generate trained neutrophils with increased cytokine production, as well as an improved ability to internalize and kill yeast cells.

### Trained neutrophils are produced *in vivo* and can defend mice against *C. albicans* infection

**Fig 4:**
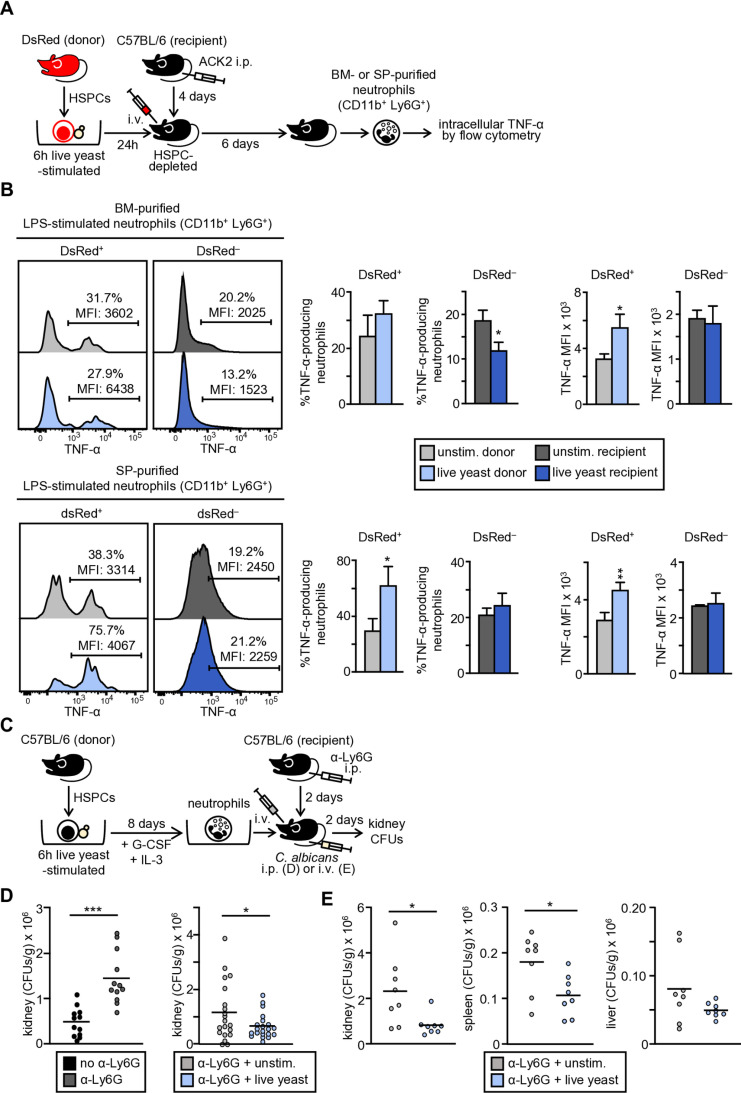
*C. albicans*-stimulated HSPCs generate trained neutrophils *in vivo* and *in vitro* differentiated trained neutrophils protect mice against infection. (A) Schematic protocol of Lin^–^ cell stimulation and transplantation (created using Power Point as a tool). Lin^–^ cells were isolated from the bone marrow of DsRed mice, *in vitro* stimulated with live *C. albicans* cells at 1:0.5 ratio (progenitor:yeast) for 6 hours, and then amphotericin B (0.5 μg/ml) was added. Lin^–^ cells were transferred to a new plate and cultured with SCF, G-CSF and IL-3 for 24 h and adoptively transferred i.v. into HSPC-depleted C57BL/6 recipient mice (injected i.p. with 500 µg/mouse of the ACK2 antibody 4 days before the adoptive transfer). 6 days later, neutrophils were purified from bone marrow and spleen for intracellular TNF-α measurement. (B) Purified neutrophils were stimulated with LPS and TNF-α production was measured in DsRed-derived (DsRed^+^) or recipient neutrophils (DsRed^–^) by intracellular flow cytometry. Histograms (1 representative experiment), indicating the % of cytokine-producing cells and MFI are shown. Bar graphs are expressed as means ± SD of pooled data from 3 independent experiments (1 recipient mouse per experiment). (C) Schematic protocol of neutrophil transplantation and infection (created using Power Point as a tool). Lin^–^ cells were cultured in the presence or absence of live *C. albicans* cells and then differentiated to neutrophils as indicated in the schematic protocol of Fig 2A. 10^6^ neutrophils were adoptively transferred i.v. into neutrophil-depleted C57BL/6 recipient mice (injected i.p. with 300 µg/mouse of the anti-Ly6G antibody 2 days before the adoptive transfer). Mice were injected i.p. with 20 x 10^6^ yeasts of *C. albicans* ATCC 26555 in 200 μl of PBS (D), or i.v. injected with 1.5 x 10^6^ yeasts of *C. albicans* PCA2 in 100 μl of PBS (E) and 2 days post-infection mice were sacrificed to assess the outgrowth of the yeasts in the internal organs. The fungal burden in kidneys, spleen and liver is expressed as CFUs per gram of tissue. (D) Results are expressed as mean ± SD of pooled data from 2 experiments (n = 6 mice each group of infected non-transplanted, and n = 10 mice each group of infected and transplanted, per experiment). (E) Results are expressed as mean ± SD of pooled data from 2 experiments (n = 4 mice each group, per experiment). Statistical significance was assessed by the Student *t* test for normally distributed data and by GLM for non-normally distributed data in one of the compared groups (kidneys). (*P < 0.05, **P < 0.01, and ***P < 0.001).

To rule out any effects due to *in vitro* differentiation, Lin^–^ cells were isolated from DsRed mice, stimulated with live *C. albicans* cells *in vitro*, and adoptively transferred to HSPC-depleted C57BL/6 recipient mice. Recipient mice were administered the ACK2 antibody, which causes HSPC death by blocking the stem cell factor (SCF) binding to c-Kit (S1B Fig). This facilitates donor HSPC engraftment and, as it does not cause inflammation, HSPC differentiation is not altered [18]. Six days later, TNF-α production following LPS stimulation was measured by intracellular flow cytometry in neutrophils purified from bone marrow and spleen (Fig 4A). Neutrophils derived from transplanted progenitors were identified as CD11b^+^ Ly6G^+^ DsRed^+^ cells whereas neutrophils from recipient mice were gated as CD11b^+^ Ly6G^+^ DsRed^–^ cells. Results revealed that bone marrow neutrophils, *in vivo* differentiated from HSPCs exposed to live *C. albicans* cells, showed a trained phenotype with an enhanced production of TNF-α in comparison with neutrophils *in vivo* differentiated from unexposed HSPCs (Fig 4B). Moreover, the *C. albicans* activation of HSPCs increased both, the percentage of cytokine-producing cells and the extent of TNF-α production by *in vivo* derived spleen neutrophils (Fig 4B). Adoptive transfer of the progenitors to mice did not alter cytokine production by neutrophils from recipient mice (Fig 4B). This indicates that HSPCs *in vitro* exposed to live *C. albicans* cells can differentiate *in vivo* to generate trained neutrophils in the bone marrow and spleen.

In order to look for a possible role of trained neutrophils in protection against infection, we adoptively transferred them to recipient mice depleted of their own neutrophils. Transplanted mice were infected intraperitoneally with *C. albicans* ATCC 26555 or intravenously with PCA2-*C. albicans* yeasts (a low virulent non-germinative strain to avoid high mortality) and fungal burden was measured in internal organs 2 days post-infection. For neutrophil depletion, recipient mice were injected intraperitoneally 2 days before transplantation with the 1A8 anti-Ly6G antibody (Figs S1C and 4C). As expected, non-transferred, Ly6G-depleted mice showed a marked increase in kidney CFUs after i.p. infection compared with control mice (non-transferred, non-Ly6G depleted) (Fig 4D). Results showed that adoptive transfer of trained neutrophils significantly decreased the fungal burden in the kidney after infection with both, the high and low virulence strains, compared with infected mice transferred with untrained control neutrophils. Moreover, transfer of trained neutrophils significantly decreased the fungal burden in the spleen after PCA2 infection, while liver CFUs were also reduced although this difference was not statistically significant (Fig 4D and 4E).

Taken as a whole, these results indicate that live *C. albicans* programs HSPCs to generate trained neutrophils *in vivo* and demonstrate the importance of trained neutrophils to defend against infection.

### The increased fungicidal activity of trained neutrophils derived from *C. albicans* treated HSPCs is conferred by mtROS

**Fig 5:**
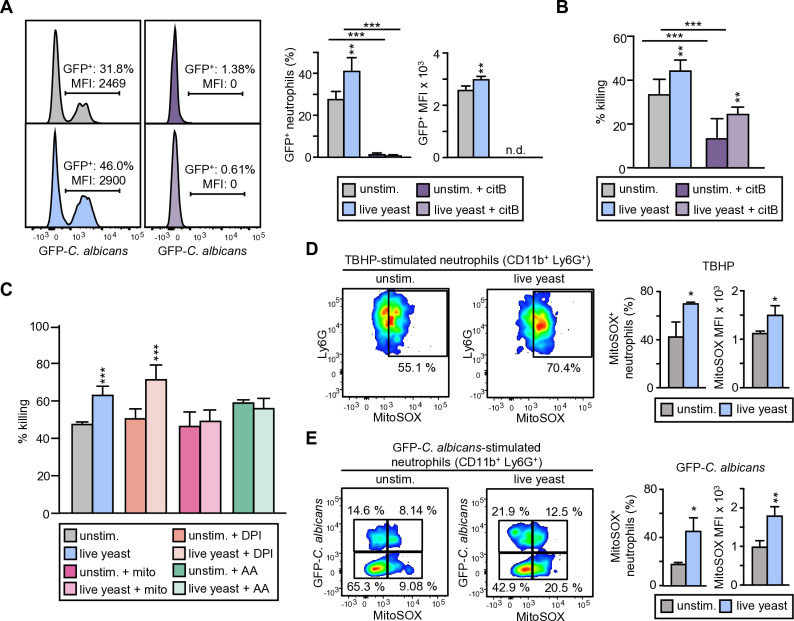
The increased fungicidal activity of trained neutrophils derived from *C. albicans*-treated HSPCs is independent of phagocytosis and is induced by mtROS production. (A) Phagocytosis assay in the presence of cytochalasin B. Neutrophils were incubated for 30 min with cytochalasin B (5 μg/ml) to inhibit their phagocytic activity. After this time, neutrophils were challenged with GFP-*C. albicans* yeasts at a 1:7.5 ratio (murine cell:yeast) for 30 min. Afterward, neutrophils were gated as Ly6G^+^ cells and the extent of phagocytosis was assessed as the percentage of GFP^+^ cells (% of phagocytosis) as well as the MFI of green fluorescence. Histograms are from 1 representative experiment, and % and MFI measurements are means ± SD from 3 independent experiments. (B) Fungicidal activity of neutrophils in the presence of cytochalasin B. Neutrophils were incubated for 30 min with cytochalasin B (5 μg/ml) to inhibit their phagocytic activity. After this time, neutrophils were challenged with viable PCA2 yeasts at a 1:3 ratio (murine cell:yeast) for 1.5 h. After incubation, samples were diluted, plated on Sabouraud dextrose agar and incubated overnight at 37 °C; CFUs were counted and killing percentages were determined as indicated in materials and methods. Triplicate samples were analyzed in each assay and expressed as means ± SD of pooled data from 3 independent experiments. (C) Fungicidal activity of DPI-, MitoTEMPO- or Antimycin A-treated neutrophils. Neutrophils were incubated for 1h with DPI (10 μM), MitoTEMPO (100 μM) or Antimycin A (1 μM) and washed away before adding the yeasts. After this time, neutrophils were challenged with viable PCA2 yeasts at a 1:3 ratio (murine cell:yeast) for 1.5 h. After incubation, samples were diluted, plated on Sabouraud dextrose agar and incubated overnight at 37 °C; CFUs were counted and killing percentages were determined as indicated in materials and methods. Triplicate samples were analyzed in each assay and expressed as means ± SD of pooled data from 2 independent experiments. (D-E) Measurement of mtROS production by flow cytometry. Neutrophils were incubated with MitoSOX-Red (1 μM) and labelled with anti-Ly6G antibody, washed and stimulated with (D) TBHP (1mM) or (E) GFP-*C. albicans* at a 1:7.5 ratio (murine cell:yeast) for 30 min. mtROS production was determined by flow cytometry as the percentage of MitoSOX-Red^+^ cells as well as the MFI of red fluorescence of Ly6G^+^ cells. Dot-plots from 1 representative experiment are shown indicating the percentage of MitoSOX-Red^+^ neutrophils (D) and the percentage of MitoSOX-Red^+^ GFP^+^ neutrophils (E). Data are presented as means ± SD of pooled data from 3 independent experiments. Statistical significance was assessed by the Student *t* test and the 1-way analysis of variance (ANOVA) followed by Dunnett test for multiple comparisons (*P < 0.05, **P < 0.01, and ***P < 0.001).

For dissecting the mechanisms by which trained neutrophils exhibited enhanced fungicidal activity, we first considered whether this is a consequence of their improved ability to internalize yeasts. As trained neutrophils showed higher expression of CD11b and it has been described that this molecule is involved in the recognition and engulfment of yeasts [11], we assessed the consequences of blocking CD11b on their ability to internalize and kill *C. albicans* cells (S1D Fig). When we performed the *C. albicans* phagocytosis assay in the presence of a functional blocking antibody or its isotype control, we found that phagocytosis was slightly reduced in both control and trained neutrophils by blocking CD11b (29.6% versus 34%, and 42.9% versus 54.6%, respectively); probably, as indicated for Dectin-1^-/-^ neutrophils, other PRRs, in our experimental conditions, are responsible for internalization of yeasts upon recognition of other PAMPs. However, trained neutrophils still displayed increased phagocytosis in the presence of the anti-CD11b antibody. Moreover, blocking CD11b had no effect on the *C. albicans* killing assay. Overall, these data suggested that the increased CD11b expression on trained neutrophils was not responsible of their increased fungicidal and phagocytic activity. To further investigate whether the increased fungicidal activity might be a consequence of the increased phagocytosis we used cytochalasin B to prevent actin polimerization for particle uptake (Fig 5A). As expected, cytochalasin B completely abolished the phagocytosis of yeasts by both, control and trained neutrophils. Moreover, neutrophils treated with cytochalasin B were significantly impaired in killing yeasts, but under these test conditions, trained neutrophils were still able to kill more *C. albicans* cells than control neutrophils, which indicated that the increased fungicidal activity was not completely dependent on phagocytosis (Fig 5B).

Neutrophil-mediated killing mechanisms of *C. albicans* include ROS production by the NADPH oxidase complex [11]. Therefore, we hypothesized that the increased fungicidal activity of trained neutrophils derived from *C. albicans* treated HSPCs might be conferred by an increased ROS production by the NADPH oxidase system. However, diphenyleneiodonium chloride (DPI) which specifically inhibits ROS production by the NADPH oxidase did not blunt neither the ability of neutrophils to kill *C. albicans* nor the increased fungicidal activity of trained neutrophils (Fig 5C). Historically, ROS production in neutrophils has been associated with NADPH oxidase, but recent work in human and mouse neutrophils suggested that mitochondria generated ROS (mtROS) enhances their bactericidal activity [19,20]. To determine if mtROS were involved in the fungicidal activity, neutrophils were treated with MitoTEMPO, a mitochondria-specific superoxide scavenger, prior to yeasts exposure. We found that treatment with mitoTEMPO decreased the fungicidal activity of trained neutrophils to match that of control neutrophils (Fig 5C). As complexes I and III are the primary sources of mitochondria-derived ROS [21] we also tested the effect of Antimycin A, an inhibitor of Complex III, and found that Antimycin A had the same impact on *C. albicans* killing by trained neutrophils than mitoTEMPO, suggesting that the mtROS are originated from Complex III (Fig 5C). To confirm these results, we directly measured mtROS using a flow cytometry-based assay using MitoSOX, a dye that specifically targets the mitochondria and fluoresces when it is oxidized by superoxide (Fig 5D and 5E). Neutrophils were treated with tert-butyl hydroperoxide (TBHP) to simulate oxidative stress, and an increased mtROS production was detected by trained neutrophils (higher % of MitoSOX^+^ neutrophils and higher MFI of total cells) (Fig 5D). To confirm that this method in fact detects ROS produced by the mitochondria, we added MitoTEMPO or Antimycin A before TBHP stimulation. We found that both inhibitors abolished the higher MitoSOX MFI detected in trained neutrophils in comparison with control neutrophils, confirming the specificity of the MitoSOX method (S1E Fig). Finally, we measured mtROS in response to live GFP-*C. albicans* cells, and we determined whether mtROS production required neutrophil phagocytosis of yeasts. We detected an increased mtROS production in both trained neutrophils directly interacting with yeasts (GFP^+^), and those that had non-internalized yeasts (GFP^–^), suggesting that mtROS are produced during infection independently of yeast contact (Fig 5E).

Finally, we investigated whether the ability to form neutrophil extracellular traps (NETs) contributed to the enhanced killing capacity of trained neutrophils. We found that trained neutrophils showed an increase in NET formation in response to both, the known NET-inducing agent PMA and *C. albicans* cells, compared to control neutrophils (S1F Fig). As expected, neutrophils treated with DPI were significantly impaired in NET formation in response to PMA, but not in response to *C. albicans* cells [11]. However, mitoTEMPO, which decreased the fungicidal activity of trained neutrophils to match that of control neutrophils, did not inhibit NET formation, nor reduced the NET formation of trained neutrophils to that of controls, clearly indicating that the increased fungicidal activity of trained neutrophils was not dependent on NET production.

Overall, these results demonstrate that HSPCs can sense live *C. albicans* cells to generate trained neutrophils that use mtROS to enhance their ability to kill yeasts.

## Discussion

Most work on trained immunity has predominantly focused on monocytes and macrophages. However, there is recent evidence that neutrophils also play important roles upon training [13]. Moreover, trained immunity does not only occur in mature myeloid cells within peripheral tissues but may in fact be initiated in their progenitors within the bone marrow (central trained immunity). HSPCs express PRRs, as well as receptors for cytokines and growth factors enabling them to recognize and respond to infections, boosting the production of myeloid cells by a process named emergency myelopoiesis [22]. In this context, our group has demonstrated that exposure of HSPCs to *C. albicans* results in the production of trained macrophages with a greater capacity to produce cytokines [6,7].

In this study, we induced neutrophil differentiation from HSPCs *in vitro* by using cytokines produced by the host specially during infection and inflammatory conditions (IL-3 and G-CSF). We then assessed whether PRR signalling on HSPCs modulates cytokine production by the neutrophils they produce. Our findings revealed that neutrophils generated from HSPCs exposed to a TLR2 agonist produce lower levels of inflammatory cytokines (tolerized neutrophils). In contrast, exposure to a Dectin-1 agonist results in the opposite phenotype, increased cytokine production (trained neutrophils). *C. albicans* yeasts, which signal via both receptors, induce higher cytokine production, indicating that combinatorial signalling by multiple receptors on HSPCs shapes their downstream immune responses. In fact, the production of TNF-α is enhanced in neutrophils generated from HSPCs exposed simultaneously to both Pam_3_CSK_4_ and depleted-zymosan compared to control neutrophils (S1G Fig). These results align with previous reports showing similar phenotypes by *in vitro* differentiated macrophages: tolerized macrophages from HSPCs exposed to Pam_3_CSK_4_ and trained macrophages from HSPCs exposed to depleted zymosan or *C. albicans* yeasts [5]. Therefore, HSPCs can be reprogrammed to produce tolerized or trained neutrophils, probably through metabolic changes and epigenetic remodelling as described in a few models for trained neutrophils. Importantly, neutrophils from BCG-vaccinated individuals present several hallmarks of trained immunity, including both epigenetic and metabolic changes [15].

To further investigate this issue, we assessed whether *C. albicans* signalling on HSPCs modulates other important anti-fungal functions of neutrophils, such as phagocytosis and killing of yeasts. We found that transient exposure of HSPCs to live yeasts, but not to inactivated yeasts, confers a higher ability to internalize and kill *C. albicans* cells to the neutrophils they produce in a Dectin-1 and TLR2-dependent manner. The use of live yeasts, which can express several virulence factors, more closely resembles the conditions under which HSPCs encounter yeasts during real infection, once the fungus reaches the bone marrow [23] or when HSPCs are mobilized to the site of infection, as it has been described in several infection models including candidiasis [7]. In this context, it was reported that intestinal colonization by *C. albicans* promotes granulopoiesis and protection against Gram-positive bacterial infection mediated by neutrophils. Interestingly, candidalysin-deficient mutant strains lost the ability to induce granulopoiesis and to protect mice against infection, highlighting the essential role of the hypha-associated virulence factor candidalysin in this model of trained neutrophils [24]. Interestingly, exposure of progenitors to live *C. albicans* cells enhances the microbicidal activity of the neutrophils derived from them, as these neutrophils have an improved ability to kill cells of *C. albicans*, *C. glabrata* and *S. aureus*. This enhanced antimicrobial function might contribute to the observed nonspecific protective effects in different models of trained immunity [9].

To explore the biological relevance of this mechanism, we used a model of HSPC transplantation to investigate their possible differentiation to trained neutrophils *in vivo*. Purified Lin^–^ cells from DsRed mice were *in vitro* stimulated with live yeasts and adoptively transferred to HSPC-depleted mice. Remarkably, under these *in vivo* conditions, we found that proinflammatory cytokine production is enhanced in DsRed neutrophils originated from *C. albicans*-stimulated HSPCs, but is not altered in DsRed neutrophils differentiated from unstimulated HSPCs or in neutrophils from recipient mice.

Going deeper, we have directly demonstrated the biological relevance of trained neutrophils in mediating resistance to infection *in vivo*. The adoptive transfer of neutrophils, generated *in vitro* from *C. albicans*-stimulated HSPCs, reduced the fungal burden in mice depleted of their own neutrophils during invasive candidiasis. While these differences in fungal burden support the notion that trained neutrophils provide protection against invasive candidiasis, a survival study would provide more definitive evidence of their role in protection. However, these experiments are technically challenging, as mice depleted of neutrophils are extremely susceptible to candidiasis, and by two days post-depletion, when the infection and adoptive transfer of trained neutrophils are performed, mice start producing their own neutrophils, potentially masking the effects of the transplanted neutrophils. Nevertheless, this result is in accordance with the improved ability to internalize and kill yeast cells of trained neutrophils generated *in vitro*, and with a previous report showing that β-glucan-induced training increases the antimicrobial activity of neutrophils, conferring prolonged protection against listeriosis [16]. Future studies should address the possibility of therapeutically administered trained neutrophils as a strategy to counteract the adverse effects of chemotherapy-induced neutropenia.

In this context, it has been described that immunization with different *Candida* species can induce granulocytic myeloid-derived suppressor cells (MDSCs) that protect against *C. albicans* infection [25–27]. However, although MDSCs express high levels of CD11b and are ROS producers, these cells are different from the trained neutrophils described in this study; MDSC-mediated protection includes abrogation of lethal inflammation whereas trained neutrophils produce higher amounts of pro-inflammatory cytokines. In fact, adoptively transferred MDSCs had no effect on fungal burden *in vivo*, whereas adoptive transfer of trained neutrophils diminished the CFUs as these cells exhibited improved ability to kill yeast cells. Therefore, although both cell-types could be protective, they do so through fundamentally different mechanisms. Further experiments to delve on the mechanisms that induce the generation of trained or MDSCs during primary infection are required to understand how host defense strategies are developed toward either host resistance (pathogen elimination) or disease tolerance (limitation of tissue damage).

We then delved into the mechanisms by which trained neutrophils exhibited improved ability to kill yeast cells. Surprisingly, the higher fungicidal activity of trained neutrophils was independent of phagocytosis, as blocking internalization with cytochalasin B significantly reduced the yeast-killing ability of both control and trained neutrophils, but it did not reduce the fungicidal activity of trained neutrophils to that of controls. Moreover, there was no correlation between the higher expression of CD11b on trained neutrophils and their higher phagocytosis or killing activity *in vitro*. However, *in vivo* higher CD11b expression might facilitate neutrophil attachment to the endothelium at sites of infection or inflammation, allowing trained neutrophils to migrate more effectively to the site of injury. Inhibition of NADPH oxidase had no effect on fungicidal activity but interestingly, MitoTEMPO significantly reduced yeast killing capacity of trained neutrophils compared to untreated trained neutrophils, indicating that production of mtROS contributes to the elevated fungicidal activity of trained neutrophils. We revealed that trained neutrophils produced significantly more mtROS than control neutrophils in response to different stimuli, but not in the presence of MitoTEMPO or Antimycin A. In addition, although trained neutrophils produce more NETs than controls, the higher fungicidal activity of trained neutrophils is independent of NETs, as MitoTEMPO significantly reduces the yeast-killing capacity of trained neutrophils, but does not reduce NET formation.

Our results suggests that HSPCs adopt an infection-responsive program that enhances oxidative phosphorylation (OXPHOS) in their derived neutrophils, thereby boosting their fungicidal activity through enhanced mtROS production. Supporting our findings, a previous study has demonstrated that trained neutrophils have enhanced antitumor activity with OXPHOS being identified as a strongly enriched pathway [15]. The potential role of OXPHOS in innate immune memory was also highlighted by suggestive associations between common single nucleotide polymorphisms in OXPHOS-related genes and the variability in the responsiveness of β-glucan- or oxidized low-density lipoprotein (oxLDL)-trained monocytes [28,29].

Production of mtROS is known to increase in macrophages following infection and has been shown to be directly involved in killing intracellular bacteria by macrophages [30,31]. More recently, mitochondria have been uncovered as a bactericidal ROS generator in neutrophils to kill *Staphylococcus aureus* and *Streptococcus pneumoniae* [19,20]. Notably, other studies have reported the involvement of mtROS in enhancing bactericidal activity of trained neutrophils generated in models of zebrafish larvae infection by *Shigella* and *Salmonella* [32,33]. In this context, Ding *et al*., described that β-glucan-trained macrophages inhibit tumour metastasis through a mechanism mediated by enhanced synthesis of sphingolipids and the subsequent accumulation of sphingosine-1-phosphate. This leads to mitochondrial fission, which is required for increased mtROS production and respiration, as well as increased cytotoxicity [34]. Additionally, a recent study also showed that oxLDL-induced trained immunity in human monocytes depends on mitochondrial function [29]. The increased mitochondrial metabolism of innate memory cells contributes to the increase in energy stores but also potentiates the production of mtROS.

Although future studies are required to define the mechanisms underlying mtROS production by trained neutrophils generated from *C. albicans*-stimulated HSPCs, our results highlight that mtROS in trained neutrophils play a key role in fungal killing. In summary, our data suggest that infection-experienced HSPCs contribute to trained immunity by providing a source of trained neutrophils with enhanced cytokine production, phagocytosis and fungicidal activity through mtROS production. Modulation of this process may help to improve innate immune responses by inducing trained neutrophils during serious infections.

## Materials and methods

### Ethics statement

All animal-related experiments were approved by the Committee on the Ethics of Animal Experiments of the University of Valencia (Permit Number: 2022/VSC/PEA/0241, 2023/VSC/PEA/0065) and performed according to Spanish law under “Reales Decretos” 1201/2005 and 53/2013. All efforts were made to minimize suffering.

### Mice

Dectin-1^–/–^ mice were purchased from The Jackson Laboratory; TLR2^–/–^ mice were provided by Dr. Shizuo Akira (Osaka University, Osaka, Japan). All knockout mice have a C57BL/6 background and were maintained at the animal production service facilities (SCSIE, University of Valencia). Wild type C57BL/6 mice (Envigo) were used as controls; the transgenic mice [B6.Cg-Tg(CAG-DsRed*MST)1Nagy/J strain, also known as DsRed.T3 (The Jackson Laboratory)] were used for *in vivo* HSPCs transplantation assays. Experiments were conducted with 8- to 12-week-old mice (regardless of gender).

### Purification of Lin^–^ cells

Mouse HSPCs were isolated as lineage-marker negative cells (Lin^–^ cells) from bone marrow. Briefly, murine bone marrow was obtained by flushing the femurs and tibias; cells were depleted of lineage positive cells by immunomagnetic cell sorting using MicroBeads (Miltenyi Biotec): bone marrow cells were labelled with a cocktail of antibodies against a panel of lineage antigens [CD5, CD45R (B220), CD11b, Gr-1 (Ly-6G/C), 7–4, and Ter-119], and then cells were purified by negative selection according to the manufacturer’s instructions. Purity of the sorted cells was assessed by labelling with anti-Lin cocktail and by flow cytometry analysis, and no Lin^+^ cells were detected.

### *In vitro* neutrophil differentiation and purification

Purified Lin^–^ cells were immediately cultured in RPMI 1640 medium supplemented with 2 mM L-glutamine, 5% heat-inactivated fetal bovine serum and 1% penicillin-streptomycin stock solution (Gibco) (hereafter complete cell culture medium) in the presence of 20 ng/ml SCF, 50 ng/ml G-CSF and 10 ng/ml IL-3 (all from Peprotech) to promote differentiation to neutrophils. Neutrophils were purified from the non-adherent fraction of the cultures by immunomagnetic cell sorting using anti-Ly6G MicroBeads UltraPure (Miltenyi Biotec).

### PAMPs and preparation of fungal stimuli

The stimuli used were depleted zymosan, Pam_3_CSK_4_, ultrapure *Escherichia coli* LPS (all from Invivogen) or inactivated *C. albicans* ATCC 26555 yeasts obtained as previously reported [35–37]. Briefly, yeasts were grown in YPD medium (1% yeast extract, 2% peptone, 2% glucose) at 28 °C up to the late exponential growth phase (A 600 nm 0.6-1), collected and washed with water. Cells were resuspended in water, and maintained for 3 h at 28 °C with shaking, and afterwards at 4 °C for 24 h (starved yeast cells). Starved yeast cells were inoculated in 5 times more volume of a minimal synthetic medium and incubated for 3 h at 28 °C. For inactivation, yeast cells were resuspended (20 x 10^6^ cells/ml) in BD Cytofix fixation buffer and incubated for 1 h at room temperature. After treatment, fungal cells were extensively washed in PBS and brought to the desired cell density in cell culture medium. Viable yeasts for *in vitro* assays were grown in YPD medium at 28 °C up to the late exponential growth phase, collected, washed in PBS, and brought to the desired cell density in cell culture medium. All procedures were performed under conditions designed to minimize endotoxin contamination as described elsewhere [37].

### Measurement of cytokine production

Neutrophils were plated in 96-well plates at a density of 50,000 cells in 200 μl of complete cell culture medium (as above described). Cells were challenged with the indicated stimuli for 24 h and cell-free supernatants were then harvested and tested for TNF-α and IL-6 release using commercial ELISA kits (eBioscience). Unstimulated neutrophils served as negative controls. Triplicate samples were analyzed in each assay.

For intracellular detection of cytokine production 200,000 *in vitro* generated neutrophils or 500,000 *in vivo* purified neutrophils were stimulated with 100 ng/ml of LPS for 4 h, with the presence of 3 μg/ml brefeldin A (eBioscence) for the final 2 h (*in vitro* generated neutrophils) or 3 h (*in vivo* purified neutrophils). Cells were first stained with antibodies against surface markers, then a cell fixation and permeabilization kit (BioLegend) and PE or BV421-labelled anti-TNF-α (clone MP6-XT22, BioLegend) were used for assessment of cytokine production by intracellular cytometry.

### *C. albicans* phagocytosis assay

For phagocytosis assay we used *C. albicans* strain ADH1G1A [38] that constitutively expresses the GFP-reporter gene, provided by Dr. Jesús Plá (Universidad Complutense, Spain). Neutrophils were plated in 24-well plates at a density of 100,000 cells in 500 μl of complete cell culture medium (as above described), and challenged with GFP-*C. albicans* at a 1:7.5 ratio (murine cell:yeast). Yeasts were settled onto the neutrophils by centrifugation and incubated for 30 min. Cells were then labelled with antibodies against surface markers and phagocytic activity was determined by flow cytometry as percentage of GFP^+^ cells as well as by the increase of the mean intensity of green fluorescence. Where indicated, the assay was performed in the presence of Cytochalasin B (Sigma) that was added at 5 μg/ml 30 min before adding the yeasts.

### Microbial killing assay

For fungicidal assay we used *C. albicans* PCA2 strain, a non-germinative strain widely used in host-fungus interactions studies [39], and *C. glabrata* CECT 1448 in order to facilitate determination of CFUs after the incubation period, as no germ tube (hyphae) aggregates are formed. Neutrophils were plated in 96-well plates at a density of 200,000 cells in 150 μl of complete cell culture medium (as above described), challenged with viable PCA2 or *C. glabrata* yeasts at a 1:3 ratio (murine cell:yeast) settled onto murine cells by centrifugation and incubated for 1.5 h. As a control, *C. albicans or C. glabrata* cells were inoculated in culture medium without murine cells. After co-incubation, samples were diluted in water, plated on Sabouraud dextrose agar, and incubated overnight at 37 °C to determine CFUs. Colonies were counted, and killing percentages were determined as follows: % killing = [1 – (CFUs sample at t = 1.5 h)/(CFUs control at t = 1.5 h)] × 100. Quintuplicate samples were analyzed in each assay.

For bacterial killing assay we used *S. aureus* CECT 4013 strain. Neutrophils were plated in 96-well plates at a density of 200,000 cells in 150 μl of cell culture medium without antibiotics, challenged with viable *S. aureus* bacteria at a 1:20 ratio (murine cell:bacteria) settled onto murine cells by centrifugation and incubated for 50 min. As a control, *S. aureus* cells were inoculated in culture medium without murine cells. After co-incubation, samples were diluted in water, plated on Mueller-Hinton agar, and incubated overnight at 37 °C to determine CFUs. Colonies were counted, and killing percentages were determined as follows: % killing = [1 – (CFUs sample at t = 50 min.)/(CFUs control at t = 50 min.)] × 100. Quintuplicate samples were analyzed in each assay.

Where indicated, the assay was performed in the presence of 10 μM DPI, or 100 μM MitoTEMPO, or 1 μM Antimycin A (all from Sigma). Inhibitors were added 1 h before the challenge and washed away before adding the yeasts.

### *In vivo* transplantation and infection experiments

For *in vivo* transplantation of HSPCs, Lin^–^ cells were isolated from DsRed mice and stimulated *in vitro* with live *C. albicans* cells at a 1:0.5 ratio (progenitor/yeast) for 6 h. After treatment, amphotericin B was added to stop fungal growth, and Lin^–^ cells were transferred to a new plate, cultured with IL-3 and G-CSF, and adoptively transferred to HSPC-depleted C57BL/6 recipient mice 24 h later. For HSPC depletion, recipient mice were injected intraperitoneally 4 days before transplantation with 500 µg of the anti-c-kit antibody ACK2, produced and purified as in [18], (the ACK2 hybridoma was a generous gift of Dr. Irving Weissman, Stanford, CA). 6 days after adoptive transfer, Ly6G^+^ cells were purified from bone marrow and spleen by immunomagnetic cell sorting and stimulated with LPS for intracellular detection of TNF-α production (as described above) by DsRed derived or recipient neutrophils.

For *in vivo* transplantation of neutrophils, Lin^–^ cells were isolated and stimulated *in vitro* with live *C. albicans* cells for 6 h (as described above). After treatment, amphotericin B was added to stop fungal growth, and Lin^–^ cells were transferred to a new plate and cultured with IL-3 and G-CSF for 8 days to promote differentiation to neutrophils. Neutrophils were purified from the non-adherent fraction of the cultures by immunomagnetic cell sorting, and 10^6^ neutrophils per mouse were adoptively transferred intravenously into neutrophil-depleted C57BL/6 recipient mice. For neutrophil depletion, recipient mice were intraperitoneally injected 2 days before transplantation with 300 µg of the anti-Ly6G antibody (clone 1A8 from BioLegend). Mice were injected intraperitoneally with 20x10^6^ yeasts of *C. albicans* ATCC 26555 in 200 μl of PBS or intravenously with 1.5x10^6^ yeasts of *C. albicans* PCA2 in 100 μl of PBS. To assess the tissue outgrowth of the microorganism, the fungal burden in the kidney, spleen and liver was determined at 2 days post-infection. The organs were weighed, homogenized in 1 ml of PBS and dilutions of the homogenates were plated on Sabouraud dextrose agar. CFUs were counted after 24h of incubation at 37 °C and expressed as CFUs per gram of tissue.

### NETs formation quantification

Neutrophils were plated in 96-well plates at a density of 100,000 cells in 100 μl of complete cell culture medium (as above described) and stimulated with PMA (Phorbol-12-myristate-13-acetate, Sigma) at 100 nM, or with *C. albicans* yeasts at a 1:7.5 ratio (murine cell:yeast) for 1h 30 min, washed and labelled with anti-Ly6G antibody. After the incubation, the cells were washed and labelled with the cell-impermeant nucleic acid probe Helix NP NIR (BioLegend) at 50 nM. NET production was determined by flow cytometry as the MFI of Helix fluorescence of Ly6G^+^ cells. Where indicated, the assay was performed in the presence of 10 μM DPI or 100 μM MitoTEMPO. Inhibitors were added 1 h before the challenge.

### Antibodies and flow cytometry analysis

Cell suspensions were labelled with various combinations of antibodies and analyzed by flow cytometry. Non-specific antibody binding was prevented by prior incubation with Fc block (anti-CD16/32). The following antibodies were used: BUV395-labelled anti-c-Kit (CD117, clone 2B8 from BD Biosciences), FITC-labelled anti-CD11b (clone M1/70 from BioLegend), APC or BV711-labelled anti-Ly6G (clone 1A8 from BioLegend), and FITC-labelled anti-Gr-1 (clone RB6-8C5 from BioLegend).

For measurement of mtROS neutrophils were plated in 96-well plates at a density of 100,000 cells in 100 μl of complete cell culture medium (as above described), and incubated with 1 μM MitoSOX-Red fluorescent dye (Invitrogen) for 15 min, labelled with anti-Ly6G antibody, washed and stimulated with GFP-*C. albicans* at a 1:7.5 ratio (murine cell:yeast), or TBHP at 1mM for 30min. mtROS production was determined by flow cytometry as the percentage of MitoSOX-Red^+^ cells as well as the MFI of red fluorescence of Ly6G^+^ cells. Flow cytometry analyses were performed on an LSR Fortessa cytometer (BD Biosciences), and data were analyzed with FlowJo 10 software.

### Statistical analysis

Statistical differences were determined using one-way analysis of variance (ANOVA) followed by Dunnett’s test for multiple comparisons and two-tailed Student’s *t*-test for dual comparisons of normally distributed data. We have used the Generalized Linear Model (GLM) for dual comparisons as statistical test when data are non-normally distributed in one of the compared groups. Data are expressed as means ± standard deviations (SDs). Significance was calculated using GraphPad Prism version 8.4.2 and R version 4.4.2 and accepted at a *P* value of < 0.05.

## Supporting information

S1 Fig (A) Flow cytometry gating of *in vitro* differentiated neutrophils from HSPCs after 8 days of culture in the presence of G-CSF and IL-3. Non-adherent cells were harvested, labelled with anti-c-Kit, anti-CD11b and anti-Ly6G, and then analyzed by flow cytometry. (B) Flow cytometry analysis of HSPCs (Lin^–^ c-Kit^+^) in the bone marrow of PBS or anti-c-Kit (ACK2) antibody injected mice (500 µg/mouse) at day 4 post-depletion. (C) Flow cytometry analysis of neutrophils (CD11b^+^ Ly6C^–^ Gr-1^+^) in the RBC-lysed blood cells of PBS or anti-Ly6G (1A8) antibody injected mice (300 µg/mouse) at day 2 post-depletion. (D) Measurement of phagocytic and fungicidal activity of CD11b-blocked neutrophils. Neutrophils were labelled with a CD11b-blocking antibody prior to the phagocytosis and killing assay. In the phagocytosis assay, neutrophils were washed and then challenged with GFP-*C. albicans* yeasts at a 1:7.5 ratio (murine cell:yeast) for 30 min. Afterward, neutrophils were gated as Ly6G^+^ cells and the extent of phagocytosis was assessed as the percentage of GFP^+^ cells (% of phagocytosis) as well as the MFI of green fluorescence. In the killing assay, neutrophils were washed and then challenged with viable PCA2 yeasts at a 1:3 ratio (murine cell:yeast) for 1.5 h. After incubation, samples were diluted, plated on Sabouraud dextrose agar and incubated overnight at 37 °C; CFUs were counted and killing percentages were determined as indicated in materials and methods. Triplicate samples were analyzed in each assay and expressed as means ± SD of pooled data from 2 independent experiments. (E) Measurement of mtROS production by MitoTEMPO- or Antimycin A-treated neutrophils. Neutrophils were incubated for 1h with MitoTEMPO (100 μM) or Antimycin A (1 μM). After this time, neutrophils were labelled with MitoSOX-Red (1 μM) and an anti-Ly6G antibody for 15 min, washed and stimulated with TBHP (1mM) for 30 min in the presence of the respective inhibitors. mtROS production was determined by flow cytometry as the MFI of red fluorescence of Ly6G^+^ cells. Data are expressed as means ± SD from 3 independent experiments. (F) NETs formation quantification. Neutrophils were incubated for 1h with DPI (10 μM), or MitoTEMPO (100 μM), stimulated with PMA at 100 nM, or with *C. albicans* yeasts at a 1:7.5 ratio (murine cell:yeast) for 1h 30 min, washed, and labelled with anti-Ly6G antibody. The cells were labelled with Helix NP NIR at 50 nM. NETs production was determined by flow cytometry as the MFI of Helix fluorescence of Ly6G^+^ cells. Data are expressed as means ± SD from 3 independent experiments. (G) Measurement of cytokine production of neutrophils derived from Lin^–^ cells exposed to TLR2 and Dectin-1 agonists, alone or simultaneously, during the first 24h. Purified neutrophils were stimulated with LPS and TNF-α production was measured by intracellular flow cytometry. Bar graphs of the % and MFI of TNF-α-producing neutrophils are shown. Duplicate samples were analyzed in the assay. Data are presented as means ± SD of data from 1 experiment. Statistical significance was assessed by the Student *t* test and the 1-way analysis of variance (ANOVA) followed by Dunnett test for multiple comparisons (*P < 0.05, **P < 0.01 and ***P < 0.001).
(TIF)

S1 File Dataset. **Quantitative data used in calculations corresponding to primary figures and to supplemental figure.** Numeric values used to generate graphs, means, and standard deviations for primary and supplemental figures are included on tabs, with each tab indicating the relevant figure panel.
(ZIP)
