# Supplementary figures and images for: Candida albicans-stimulated hematopoietic stem and progenitor cells generate trained neutrophils with enhanced mitochondrial ROS production that defend against infection
Source: PLoS Pathog. 2025 May 13;21(5):e1013170. doi: 10.1371/journal.ppat.1013170 (PMC12101778; doi:10.1371/journal.ppat.1013170)

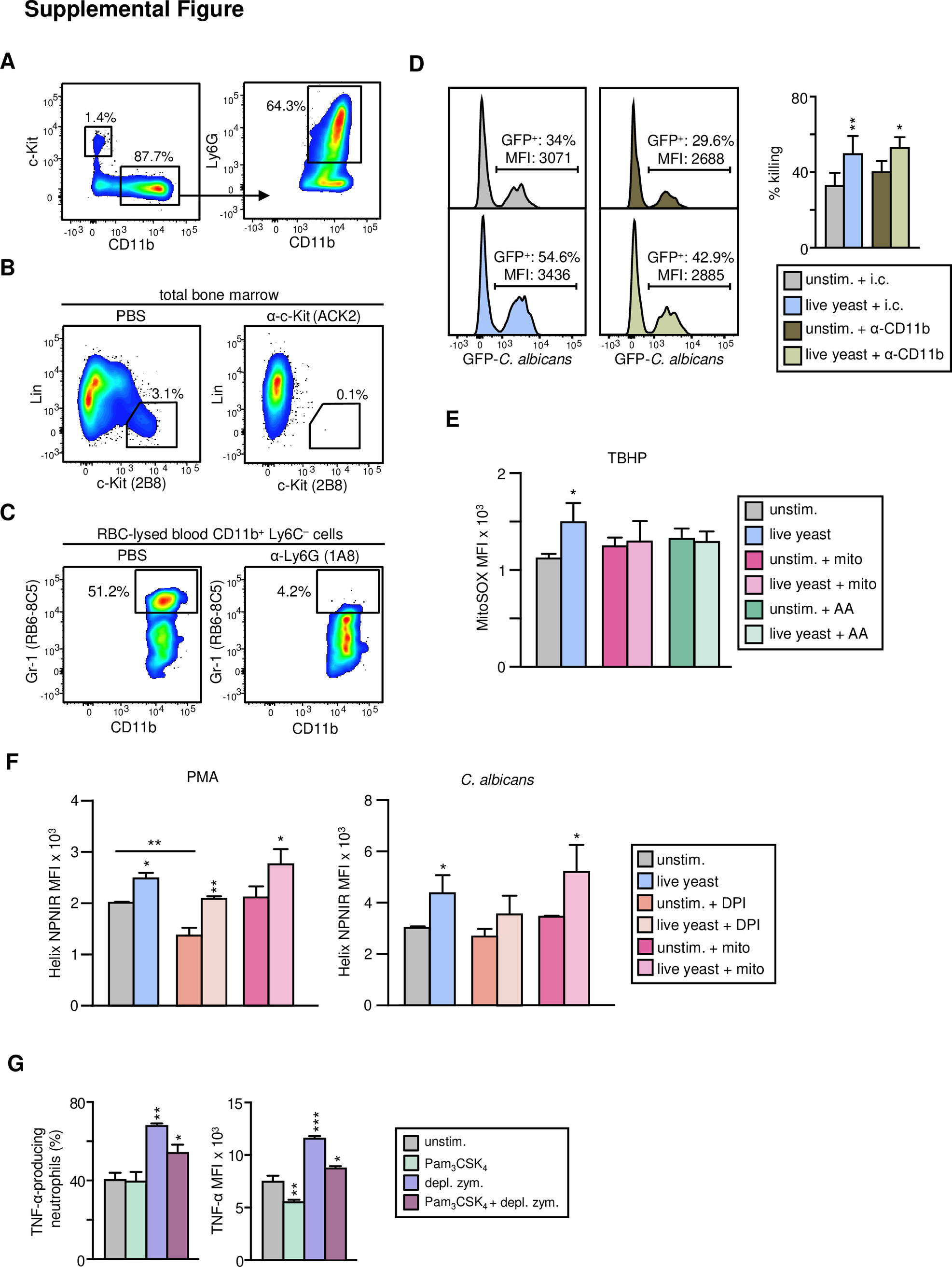

Supplement: S1 Fig — Non-adherent cells were harvested, labelled with anti-c-Kit, anti-CD11b and anti-Ly6G, and then analyzed by flow cytometry. (B) Flow cytometry analysis of HSPCs (Lin– c-Kit+) in the bone marrow of PBS or anti-c-Kit (ACK2) antibody injected mice (500 µg/mouse) at day 4 post-depletion. (C) Flow cytometry analysis of neutrophils (CD11b+ Ly6C– Gr-1+) in the RBC-lysed blood cells of PBS or anti-Ly6G (1A8) antibody injected mice (300 µg/mouse) at day 2 post-depletion. (D) Measurement of phagocytic and fungicidal activity of CD11b-blocked neutrophils. Neutrophils were labelled with a CD11b-blocking antibody prior to the phagocytosis and killing assay. In the phagocytosis assay, neutrophils were washed and then challenged with GFP-C. albicans yeasts at a 1:7.5 ratio (murine cell:yeast) for 30 min. Afterward, neutrophils were gated as Ly6G+ cells and the extent of phagocytosis was assessed as the percentage of GFP+ cells (% of phagocytosis) as well as the MFI of green fluorescence. In the killing assay, neutrophils were washed and then challenged with viable PCA2 yeasts at a 1:3 ratio (murine cell:yeast) for 1.5 h. After incubation, samples were diluted, plated on Sabouraud dextrose agar and incubated overnight at 37 °C; CFUs were counted and killing percentages were determined as indicated in materials and methods. Triplicate samples were analyzed in each assay and expressed as means ± SD of pooled data from 2 independent experiments. (E) Measurement of mtROS production by MitoTEMPO- or Antimycin A-treated neutrophils. Neutrophils were incubated for 1h with MitoTEMPO (100 μM) or Antimycin A (1 μM). After this time, neutrophils were labelled with MitoSOX-Red (1 μM) and an anti-Ly6G antibody for 15 min, washed and stimulated with TBHP (1mM) for 30 min in the presence of the respective inhibitors. mtROS production was determined by flow cytometry as the MFI of red fluorescence of Ly6G+ cells. Data are expressed as means ± SD from 3 independent experiments. (F) NETs formation q [file ppat.1013170.s001.tif]
